# The combination of malnutrition-inflammation and functional status limitations is associated with mortality in hemodialysis patients

**DOI:** 10.1038/s41598-020-80716-0

**Published:** 2021-01-15

**Authors:** Eiichiro Kanda, Marcelo Barreto Lopes, Kazuhiko Tsuruya, Hideki Hirakata, Kunitoshi Iseki, Angelo Karaboyas, Brian Bieber, Stefan H. Jacobson, Indranil Dasgupta, Bruce M. Robinson

**Affiliations:** 1 Medical Science, Kawasaki Medical School, 577 Matsushima, Kurashiki, Okayama 701-0192 Japan; 2 Arbor Research Collaborative for Health, Ann Arbor, MI USA; 3 Department of Nephrology, Nara Medical University, Nara, Japan; 4 Fukuoka Renal Clinic, Fukuoka, Japan; 5 Nakamura Clinic, Okinawa, Japan; 6 Division of Nephrology, Department of Clinical Sciences, Karolinska Institutet, Danderyd University Hospital, Stockholm, Sweden; 7 Heartlands Hospital Birmingham, Birmingham, UK; 8 Warwick Medical School, University of Warwick, Coventry, UK

**Keywords:** End-stage renal disease, Haemodialysis

## Abstract

The identification of malnutrition-inflammation-complex (MIC) and functional status (FS) is key to improving patient experience on hemodialysis (HD). We investigate the association of MIC and FS combinations with mortality in HD patients. We analyzed data from 5630 HD patients from 9 countries in DOPPS phases 4–5 (2009–2015) with a median follow-up of 23 [IQR 11, 31] months. MIC was defined as serum albumin < 3.8 g/dL and serum C-reactive protein > 3 mg/L in Japan and > 10 mg/L elsewhere. FS score was defined as the sum of scores from the Katz Index of Independence in Activities of Daily Living and the Lawton-Brody Instrumental Activities of Daily Living Scale. We investigated the association between combinations of MIC (+/−) and FS (low [< 11]/high [≥ 11]) with death. Compared to the reference group (MIC−/high FS), the adjusted hazard ratios [HR (95% CI)] for all-cause mortality were 1.82 (1.49, 2.21) for MIC−/low FS, 1.57 (1.30, 1.89) for MIC+/high FS, and 3.44 (2.80, 4.23) for MIC+/low FS groups. Similar associations were observed with CVD-related and infection-related mortality. The combination of MIC and low FS is a strong predictor of mortality in HD patients. Identification of MIC and poor FS may direct interventions to lessen adverse clinical outcomes in the HD setting.

## Introduction

The association between measures of protein-energy malnutrition and inflammation in dialysis patients with adverse outcomes is strong^1–3^. Furthermore, it is common for these two conditions to occur concomitantly. Due to these circumstances, researchers have often used the term malnutrition-inflammation complex (MIC) to designate the combination of the two conditions in this population^4^. Known risk factors for mortality in the general population (e.g., high BMI, high total serum cholesterol) are less frequent in MIC patients and yet they have higher risk of death due to their pro-inflammatory state^1^.

MIC is also associated with decreased body stores of protein and energy fuels^5,6^, affecting up to half of hemodialysis (HD) patients^7^ and ultimately leading to sarcopenia and frailty^5,6,8^. The initiation of HD is also associated with a decline in functional status (FS) in the elderly^9^, leading to a vicious cycle of reduced food intake, due to decreased physical function and lack of appetite, resulting in worsened nutritional status^1^. The Dialysis Outcomes and Practice Patterns Study (DOPPS) showed that low activities of daily living (ADL) is associated with mortality in hemodialysis patients^10^.

Several national and international guidelines recommend both nutritional and physical therapy for dialysis patients to improve their experience and outcomes^11–13^. Prevention through early identification of patients with MIC, along with the appropriate therapeutic interventions, are important actions to consider in order to improve the patient experience on HD^6,14,15^.

In this study, we explore the combined effects of MIC and FS on mortality in HD patients.

## Results

**Figure 1:**
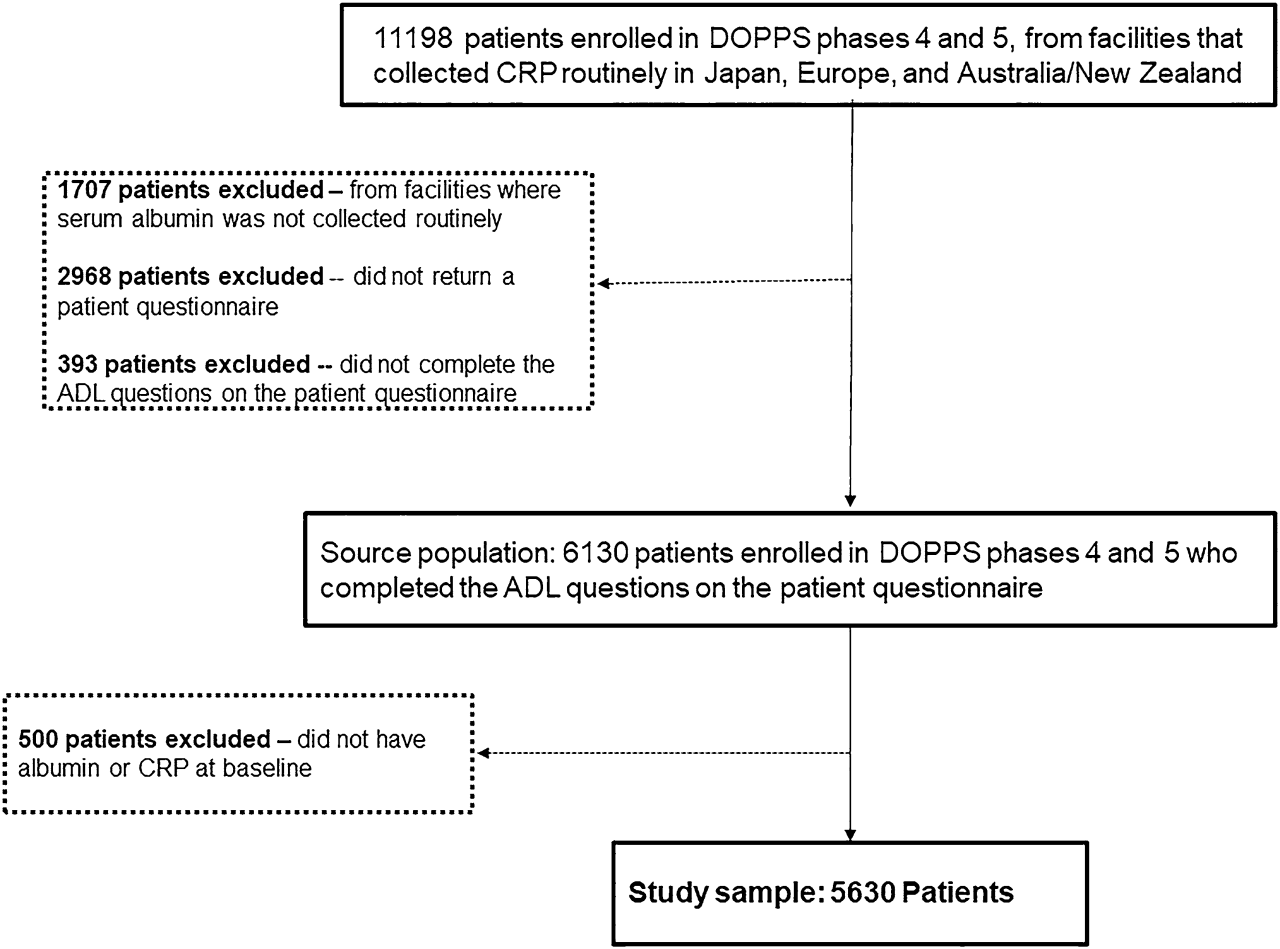
STROBE diagram of the study sample. ADL, activities of daily living; CRP, C-reactive protein.

There were 15,266 patients enrolled in DOPPS phases 4 and 5 from facilities that collected CRP routinely in Japan, Europe, and Australia/New Zealand. We excluded patients from facilities that did not often collect albumin (N = 1707). We also excluded patients who did not return a PQ (N = 2968) and those who did not complete the FS assessment (N = 393). Among eligible facilities, we also excluded patients who did not have albumin and/or CRP levels measured at study baseline (N = 500). Our study population consisted of 5630 HD patients with a median follow-up of 23 [IQR 11, 31] months (see STROBE diagram, Fig. 1). Japanese clinics accounted for 42% of patients in our sample, European clinics for 53% and clinics from Australia/New Zealand for 5%. There were 869 deaths over the follow-up period (crude death rate = 8.9 per 100 patient-years), 301 deaths reported with a CV cause (3.1 per 100 patient-years), and 136 deaths due to an infectious cause (1.4 per 100 patient-years).

**Figure 2:**
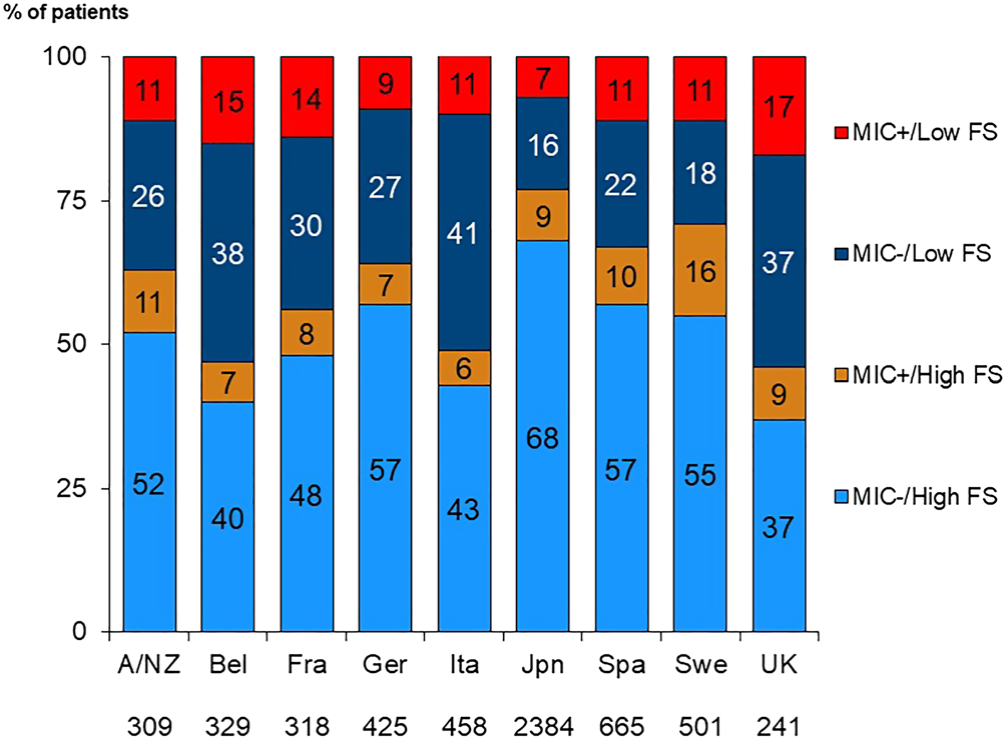
Distribution of MIC and FS combinations by country. MIC, malnutrition-inflammation-complex; FS, functional status; A/NZ, Australia/ New Zealand; Bel, Belgium; Fra, France; Ita, Italy; Jpn, Japan; Spa, Spain; Swe, Sweden; UK, United Kingdom.

High CRP was present in 27% of the population (Table 1), ranging from 23% in Japan to 39% in the UK. Low albumin was present in 53% of patients and varied by country: 39% in Spanish patients and as high as 74% of patients in Sweden. MIC was present in 19%, and low FS was observed in 33% of our sample (Table 2). The prevalence of the combinations of MIC (+/−) and FS (low/high) by country is shown in Fig. 2. Japan had the highest prevalence (68%) of patients without any conditions (MIC−/high FS), whereas the prevalence of both (MIC+/low FS) was highest in the UK (17%).

**Table 1:** Percentage of the patients with MIC.

CRP categories	Albumin categories		
	< 3.8 g/dL	≥ 3.8 g/dL	Total
Low CRP	1915 (34%)	2200 (39%)	4213 (73%)
High CRP	1054 (19%)	461 (8%)	1558 (27%)
Total	2969 (53%)	2661 (47%)	5630

Proportions (%) calculated with the total (N = 5630) as the denominator. High serum CRP level was defined as greater than 3 mg/L in Japan and 10 mg/L in other DOPPS countries.

MIC, malnutrition-inflammation-complex; CRP, C-reactive protein.

**Table 2:** Distribution of functional status (FS) categories and MIC in the study population.

FS categories	N (%)	MIC categories	N (%)
High FS	3774 (67%)	MIC−	4576 (81%)
Low FS	1856 (33%)	MIC+	1054 (19%)
Total	5630	Total	5630

Proportions (%) calculated with the total (N = 5630) as the denominator. Low FS defined as the sum of scores from the self-reported limitations in the Katz Index of Independence in Activities of Daily Living (range 0–5) and the Lawton-Brody Instrumental Activities of Daily Living Scale (range 0–8) less than 11.

MIC, malnutrition-inflammation-complex; FS, functional status.

Patient characteristics by MIC and FS combinations are shown in Table 3. Patients in the MIC−/high FS group were much younger (61.1 vs. 71.2), and had lower prevalence of comorbid conditions compared to patients with MIC+ and low FS. The prevalence of heart disease comorbidities was higher in patients who had the combination of MIC+ and low FS, as coronary artery disease was present in 46% of patients with MIC+/low FS and only 25% of patients with MIC−/high FS. Moreover, the prevalence of heart failure was higher in patients with MIC+/low FS (28%) than MIC−/high FS (16%). MIC+/low FS patients also had lower mean creatinine (7.2 mg/dL vs. 9.9 mg/dL), phosphorus values (4.7 vs. 5.2 mg/dL), and a greater use of catheter as vascular access for HD (30% vs. 11%) than MIC−/high FS.

**Table 3:** Patient characteristics according to combined MIC/FS categories.

Patient characteristics	MIC−/high FS	MIC+/high FS	MIC−/low FS	MIC+/low FS
Patients, n (row %)	3259 (58%)	515 (9%)	1317 (23%)	539 (10%)
**Demographics**				
Age (years)	61.1 (13.8)	65.1 (12.4)	71.2 (11.9)	71.2 (11.5)
Male (%)	64%	70%	56%	62%
Body Mass index (kg/m^2^)	23.7 (5.1)	24.2 (5.8)	24.6 (5.5)	25.1 (6.7)
Median time on dialysis [IQR] (years)	3.1 [0.9, 8.3]	3.2 [0.8, 7.9]	2.8 [0.9, 6.3]	2.7 [0.8, 6.8]
Urine volume > 200 ml	35%	28%	30%	27%
**Comorbidities**				
Diabetes (%)	31%	36%	49%	47%
Hypertension (%)	85%	85%	83%	79%
Coronary artery disease (%)	26%	36%	43%	45%
Congestive heart failure (%)	16%	22%	28%	28%
Other heart disease (%)	24%	32%	38%	40%
Cerebrovascular disease (%)	9%	12%	24%	22%
Cancer (%)	13%	16%	16%	21%
Psychiatric disorder (%)	10%	13%	16%	19%
Peripheral vascular disease (%)	16%	26%	36%	44%
Recurrent cellulitis (%)	4%	6%	12%	18%
Lung disease (%)	7%	12%	13%	17%
**Laboratory**				
Median CRP [IQR] (mg/L)	1.9 [0.7, 5.0]	15.0 [10.0, 28.5]	3.1 [1.0, 6.1]	21.0 (12.0, 39.6)
WBC (1000*cells/mm^3^)	6.3 (2.0)	7.2 (2.51)	6.5 (2.1)	7.4 (2.8)
Albumin (g/dL)	3.84 (0.40)	3.35 (0.33)	3.70 (0.43)	3.19 (0.42)
Hemoglobin (g/dL)	11.2 (1.3)	10.8 (1.4)	11.2 (1.3)	10.8 (1.5)
Creatinine (mg/dL)	9.9 (3.0)	8.8 (2.7)	7.8 (2.5)	7.2 (2.3)
Phosphorus (mg/dL)	5.2 (1.5)	5.1 (1.6)	4.8 (1.4)	4.7 (1.6)
Total calcium (mg/dL)	9.0 (0.7)	8.8 (0.7)	9.0 (0.7)	8.8 (0.8)
**Vascular access**				
Catheter use (%)	11%	17%	26%	30%

Values are shown as average (standard deviation).

MIC, malnutrition-inflammation-complex; FS, functional status; BMI, body mass index; GI, gastrointestinal; CRP, C-reactive protein; IQR, interquartile range; WBC, white blood cell count.

As individual predictors, MIC+ and low FS were each associated with an increased risk of death (HR for MIC+: 2.11 [1.84, 2.42]; HR for low FS: 1.76 [1.50, 2.07]). There was no interaction between MIC and low FS in the association with mortality (p for interaction = 0.40). Estimated effects of MIC/FS combinations on all-cause mortality are provided by level of covariate adjustment in Table 4. The unadjusted associations were very strong (Model 1), and remained robust after adjustment for possible confounders (Model 4). The adjusted hazard ratios (95% CI) for all-cause mortality, compared to the reference group of MIC−/high FS were 1.82 (1.49, 2.21) for MIC−/low FS, 1.57 (1.30, 1.89) for MIC+/high FS, and 3.44 (2.80, 4.23) for MIC+/ low FS.

**Table 4:** Association of MIC and FS status combinations on risk of death, by level of adjustment.

	N (%)	Model 1	Model 2	Model 3	Model 4
MIC−/high FS	3259 (58)	1 (ref)	1 (ref)	1 (ref)	1 (ref)
MIC−/low FS	515 (9)	2.81 (2.33, 3.38)	2.20 (1.81, 2.68)	2.07 (1.70, 2.52)	1.82 (1.49, 2.21)
MIC+/high FS	1317 (23)	2.54 (2.07, 3.13)	2.04 (1.69, 2.46)	1.71 (1.41, 2.08)	1.57 (1.30, 1.89)
MIC+/low FS	539 (10)	6.98 (5.74, 8.49)	5.25 (4.34, 6.35)	4.27 (3.48, 5.24)	3.44 (2.80, 4.23)

Crude death rate was 8.9 per 100 patient-years (N = 869 deaths). The association of MIC and FS status with risk of death was evaluated using Cox proportional hazards models adjusted for baseline characteristics. The models were defined by the covariates added to the model, as follows:

**Model 1**: stratified by country and phase of the study.

**Model 2**: Adjusted for age, sex, and body mass index.

**Model 3**: Model 2 adjustments, plus comorbidity history, and catheter use.

**Model 4**: Model 3 adjustments, plus serum creatinine and phosphorus levels, WBC count, hemoglobin level, and vintage (main model).

Values are hazard ratios (95% confidence interval).

MIC, malnutrition-inflammation-complex; FS, functional status.

The cause-specific analysis for CV and infectious mortality followed a similar pattern of association (Table 5), with MIC−/high FS patients having the lowest risk and patients with MIC+/low FS with a nearly four-fold risk for infection-related (HR: 3.91 [2.35, 6.51]) and for CVD-related mortality (HR: 3.97 [2.71, 5.83]). There were no interactions of MIC and FS for CV or infectious mortality (p for interaction = 0.10 and 0.19 for CV and infectious mortality, respectively).

**Table 5:** Combined effects of MIC and FS status on risk of cause-specific death.

	Infection-related mortality	CVD-related mortality
MIC−/high FS	1 (ref)	1 (ref)
MIC−/low FS	1.52 (0.83, 2.79)	1.59 (1.06, 2.38)
MIC+/high FS	1.53 (0.91, 2.56)	1.49 (1.07, 2.06)
MIC+/low FS	3.91 (2.35, 6.51)	3.97 (2.71, 5.83)

The association of MIC and FS status with risk of cause-specific death was evaluated using Cox proportional hazards with models adjusted with same covariates as model 3 in Table 4. Results are shown as hazard ratio (95% confidence interval). Infection related death rate was 1.4/100 patient-years (total of 136 deaths) and CVD-related death rate was 3.1/100 patient-years (total of 301 deaths), respectively.

MIC, malnutrition-inflammation-complex; FS, functional status.

## Discussion

This analysis of international DOPPS data shows that MIC in combination with low FS is associated with a high risk of death in HD patients. The presence of one of the components increases the risk by 56% (low FS only) to 75% (MIC+ only). The presence of both conditions results in a more than three-fold higher rate of all-cause mortality, and a nearly four-fold higher rate of CVD- and infection-related death. To the best of our knowledge, this is the first study reporting the combined impact of MIC and low FS in HD patients.

Although there was higher prevalence of diabetes, CVD, and cancer in the MIC+/low FS group, the higher risk of all-cause and cause-specific death persisted even after extensive adjustment for these and other risk factors. Previous observational studies indicated that the inverse association between serum cholesterol and mortality in HD patients, paradoxical in comparison to the general population, is likely due to malnutrition and inflammation^5,16^. These lines of evidence suggest that malnutrition and systemic inflammation may play important roles in increasing the risk of death in HD patients.

The MIC+/low FS patients had lower mean serum creatinine and phosphorus levels, indicating low muscle mass and protein intake which, along with low FS, represent risk factors for death in dialysis patients^17^. Interventions to improve nutritional status also improve inflammation, as well as physical performance. A randomized controlled trial (RCT) in which HD patients received oral supplementation with a whey protein, soy protein, or placebo beverage for 6 months before each HD session showed that protein supplementation reduced predialysis interleukin 6 levels and CRP, with gait speed and shuttle walk test improvement observed, as well^18^. An observational study of an oral nutritional supplement prescription for patients with albumin ≤ 3.5 g/dL during HD showed an association with reduced mortality despite no change in serum albumin levels^19^. In our study, patients with both MIC+ and low FS had a much greater risk of death due to CV and infection causes than patients with only 1 of the 2 exposures, which is suggestive of a potential biological interaction in the CKD setting.

A systematic review and meta-analysis suggested that physical exercise, including during the HD session, improves physical functioning, cardiovascular fitness, health related quality of life, oxygen uptake (VO2-max), and dialysis adequacy (KT/V) in HD patients^20^. Furthermore, protein anabolism is enhanced by resistance training before HD and nutrition intake during dialysis^21^. A RCT showed that a 6-min walking exercise on a treadmill during a dialysis session combined with nutritional supplements is a feasible and well accepted strategy that improves physical function and quality of life^22^. Therefore, the combined use of nutritional intervention and exercise training may be effective in improving prognosis of MIC and low FS in HD patients such as the ones participating in this study. The Japanese Society of Renal Rehabilitation, as well as other international guidelines, highlight the importance of both nutritional therapy and exercise interventions for patients with PEW^11^.

DOPPS has shown differences in the mortality rates and practice patterns that affect HD patients’ outcomes between the participating countries^23^. For instance, the first-year mortality rate in Belgium is greater than in Japan or the United Kingdom^23^. Our study showed that MIC+/low FS is an independent risk factor for death and was less prevalent in Japan compared to other countries, which might in part explain the lower mortality in Japanese HD patients. On the other hand, similar distributions of MIC+/low FS were found in the United Kingdom and Belgium. DOPPS has also shown that dialysis catheter use is preferred by patients in Belgium, and the rate of catheter use is higher in Belgium than in other countries^24^. The use of catheters for vascular access in HD is associated with malnutrition and inflammation, as well as a higher risk of death^25^. Thus, HD-related factors other than MIC+/low FS, such as catheter use, might lead to the difference in mortality across DOPPS countries. However, this analysis was adjusted for catheter use, although residual confounding cannot be completely excluded.

This study has some limitations. Although causal inference cannot be made due to the observational nature of this study, this analysis may be adequate in accomplishing its objective of showing a higher risk of death associated with having these nutritional and inflammation risk factors. We also acknowledge the potential for misclassification of causes of death, which may bias the estimated associations with CVD and infection-related mortality. A clinical trial on MIC/low FS would demand an elaborate intervention that may not have an impact on the outcomes. The use of real-world data from a great number of in-center hemodialysis center patients and standardized protocol for data collection are important strengths of this study. Given the random sampling design of the DOPPS, our study sample can be viewed as representative of the HD population in each participating country, and the large sample size provided sufficient statistical power to detect the proposed associations^26^. Third, nutrition intake is influenced by food. However, because DOPPS data does not include diet record, we could not evaluate the effects of diet on nutritional status. Fourth, Kanda E. et al. showed the importance and the cutoff levels of serum creatinine levels as a nutritional index using a database of the Japanese Society for Dialysis Therapy^8^. However, it was difficult to use serum creatinine levels for the categorization of MIC, because three indices might categorize patients into many groups and complicate study design. A simple and accurate index for malnutrition is one of the important themes to improve nutritional conditions of dialysis patients.

## Conclusion

This study demonstrates that the combination of MIC and low FS is associated with a higher risk of both all-cause and cause-specific deaths in HD patients. This suggests that the diagnosis of malnourishment and inflammation with albumin and CRP, as well as FS, may be useful for risk stratification for survival in these patients. We believe that this approach will direct attention to nutritional and exercise interventions, based on simple and objective measures, to improve experience and outcomes of patients receiving HD.

## Materials and methods

### Data source

DOPPS is a prospective cohort study, ongoing since 1996, involving center-based adult chronic HD patients in more than 20 countries. Study sites and patients are randomly selected to achieve national samples. Detail on the study design is included in previous publications^16,27^ and at http://www.DOPPS.org. DOPPS maintains institutional review board or ethical committee approvals in all participating countries, and informed consent is collected from patients selected for study participation. This work included a cohort of HD patients from Australia, France, Germany, Italy, Japan, New Zealand, Spain, Sweden, and the United Kingdom, enrolled in DOPPS phases 4 (2009–2011) and 5 (2012–2015).

### Measures

We defined MIC by the presence of both low albumin and high C-reactive protein (CRP) levels. We considered low albumin as serum albumin levels < 3.8 g/dL. Because CRP levels are systematically lower in Japan, two thresholds for high CRP level were defined: > 3 mg/L in Japan and > 10 mg/L elsewhere^28^. The distribution of CRP > 3 mg/L in Japan is still lower (prevalence of 23%) than the prevalence of CRP > 10 mg/L in other DOPPS countries (prevalence ranges from 24% in Italy to 39% in the UK).

FS was assessed by the DOPPS self-reported patient questionnaire (PQ), and details were described in the previous study^10^. In brief, patients indicated their level of ability to perform five activities of daily living tasks (Katz questionnaire: eating, getting dressed, bathing, using the toilet, transferring from bed to chair) and eight instrumental activities of daily living tasks (Brody questionnaire: using the telephone, getting to places beyond walking distance, grocery shopping, preparing meals, doing housework or handyman work, doing laundry, taking medications, and managing money)^29,30^. Both questionnaires have been validated in the general population. Low FS was defined by a FS score of < 11 out of the 13-points of the combined Katz and Brody scales, calculated as in Jassal et al.^10^ on which a score of 13 represents full functional independence. Time-to-event outcomes included all-cause mortality (primary) and cause-specific death (secondary) due to (1) CVD or (2) infection. We explored both the main effects and the combined effect between the two exposure variables on mortality.

### Patient/study sample

Patients were included if they answered all 13 FS questions on the PQ and had available data on serum albumin and CRP in the three months before PQ completion. We limited our sample to facilities that routinely measured CRP and albumin levels in over half of their patients to limit cases where they were measured “by indication”. We excluded patients with a prior history of limb amputation to accurately evaluate BMI.

### Statistical analyses

Cox proportional hazards models were used to analyze the association of the combinations of MIC (+/−) and FS (low/high) with mortality, stratified by country and DOPPS phase, and accounting for facility clustering using robust sandwich covariance estimators. Models were adjusted for case-mix (patient characteristics: age, sex, body mass index, vintage, and comorbid history of diabetes mellitus, hypertension, coronary artery disease, congestive heart failure, other heart diseases, cerebrovascular disease, cancer, gastrointestinal bleeding, psychiatric disorder, peripheral vascular disease, recurrent cellulitis, and lung disease), central venous catheter use, and laboratory values (serum creatinine and phosphorus levels, white blood cell count [WBC], and hemoglobin level). Time at risk started at the completion of the PQ and ended at the time of death, 7 days after leaving the facility due to transplant or transfer, 7 days after changing modality, loss to follow-up, or administrative study end. The proportional hazards assumption was confirmed. Additionally, cause-specific Cox models were used to test the association of MIC and/or FS with cause-specific (infection, CVD-related) mortality. Deaths by other causes were censored in this approach.

Overall, missingness for covariates was low (< 20% for the majority of covariates). For missing data, we used the sequential regression multiple imputation method implemented by IVEware^31^, and the MIAnalyze procedure in SAS/STAT 9.4. All analyses were carried out using SAS software, version 9.4 (SAS institute, Cary, NC).

## Data Availability

The data that support the findings of this study are available from Arbor Research Collaborative for Health, but restrictions apply to the availability of these data which were used for the current study, and so are not publicly available. Data are however available from the authors upon reasonable request and with permission of Arbor Research Collaborative for Health.
